# Loss-of-rescue of *Ryr1*^I4895T^-related pathology by the genetic inhibition of the ER stress response mediator CHOP

**DOI:** 10.1038/s41598-022-25198-y

**Published:** 2022-11-30

**Authors:** Serena Germani, Alessia Celeste Marchetti, Andrea Guidarelli, Orazio Cantoni, Vincenzo Sorrentino, Ester Zito

**Affiliations:** 1 Istituto di Ricerche Farmacologiche Mario Negri IRCCS, Via Mario Negri 2, 20156 Milan, Italy; 2 Department of Biomolecular Sciences, University of Urbino Carlo Bo, Urbino, Italy; 3 Department of Molecular and Developmental Medicine, University of Siena, Siena, Italy

**Keywords:** Cell biology, Mechanisms of disease

## Abstract

*RYR1* is the gene encoding the ryanodine receptor 1, a calcium release channel of the endo/sarcoplasmic reticulum. I4898T in *RYR1* is one of the most common mutations that give rise to central core disease (CCD), with a variable phenotype ranging from mild to severe myopathy to lethal early-onset core-rod myopathy. Mice with the corresponding I4895T mutation in *Ryr1* present mild myopathy when the mutation is heterozygous while I4895T homozygous is perinatal-lethal. Here we show that skeletal muscles of I4895T homozygous mice at birth present signs of stress of the endoplasmic reticulum (ER stress) and of the related unfolded protein response (UPR) with increased levels of the maladaptive mediators CHOP and ERO1. To gain information on the role of CHOP in the pathogenesis of RYR1^I4895T^-related myopathy, we generated compound *Ryr1*^I4895T^, *Chop* knock-out (-/-) mice. However, the genetic deletion of *Chop,* although it attenuates ER stress in the skeletal muscle of the newborns, does not rescue any phenotypic or functional features of *Ryr1*^I4895T^ in mice: neither the perinatal-lethal phenotype nor the inability of *Ryr1*^I4895T^ to respond to its agonist caffeine, but protects from ER stress-induced apoptosis. These findings suggest that genetic deletion of the ER stress response mediator CHOP is not sufficient to counteract the pathological *Ryr1*^I4895T^ phenotype.

## Introduction

Three distinct genes encode three isoforms of the ryanodine receptor (RYR1, RYR2, and RYR3) which have different tissue distributions: RYR1 is enriched in the skeletal muscle, RYR2 in cardiac muscle, and RYR3 is expressed ubiquitously^1–3^. All three RYRs are calcium release channels, localized in the membrane of the endoplasmic reticulum (ER) and/or sarcoplasmic reticulum (SR). RYR1 is involved in the mechanism of excitation–contraction (E-C) coupling in skeletal muscle^4^. This implies that an action potential travels through the transverse (T)-tubule triggering the physical interaction between the dihydropyridine receptor (DHPR) and RYR1, which results in Ca^2+^ release from SR/ER, hence contraction of muscle fibers^4^.

Mutations in *RYR1* can be either homozygous or heterozygous and can lead to distinct channel defects, i.e. leaky, E-C uncoupling, and loss of RYR1 channel. These mutations can give rise to many debilitating rare myopathies ranging from central core disease (CCD, OMIM n. 117,000), to multiminicore disease (MmD, OMIM n. 602,771), to nemaline rod myopathy (OMIM n. 161,800), and including life-threatening malignant hyperthermia (OMIM n.145,600)^5–8^.

I4898T mutation is one of the most common mutations in *RYR1*, and results in a variable phenotype ranging from mild to severe CCD to the lethal early-onset core-rod myopathy^9^. Mice knock-in for R*yr1*^I4895T^, which corresponds to I4898T in humans, show a mild myopathic phenotype in the C57BL/6 background when the mutation is in heterozygosity while it is perinatal-lethal when the mutation is in homozygosity^10^. Functional studies of R*yr1*^I4895T^ suggest that this mutation falls into the category of an E-C uncoupling receptor, meaning RYR1 is not responsive to electrical or agonist stimulation^11^.

The mild myopathy in heterozygous mutant mice (R*yr1*^I4895T/+^) is associated with progressive ER stress and UPR and increased levels of the mediators CHOP (CAATT enhancer-binding protein homologous protein) and ERO1-alpha (Endoplasmic reticulum oxidoreductin 1-alpha, henceforth ERO1). Importantly, treatment with the chemical chaperone 4-PBA rescues this pathological muscle phenotype while it attenuates ER stress, suggesting that ER stress/UPR might be an important pathogenic component of this RYR1-related myopathy^10^.

During UPR, CHOP and its downstream targets ERO1 and GADD34 might be mediators of the maladaptive branch of this response given their ability to generate reactive oxygen species (ROS) and to restart the protein synthesis  respectively in condition of altered proteostasis thereby triggering apoptotic stimuli. Indeed, in some cases, deletion of the gene encoding CHOP preserves tissue function in case of chronic ER stress/maladaptive UPR^12–14^. In the field of genetic myopathies, ablation of CHOP rescued the muscle pathological phenotype of a mouse model with loss of function of *SepN1*, another gene whose mutations are associated with MmD^15–18^. Thus, to test whether CHOP is also an important contributor to the *Ryr1*^I4895T^ muscle phenotype we engineered a mouse model lacking *Chop* and expressing *Ryr1*^I4895T^. Although skeletal muscles of *Ryr1*^I4895T^ at birth show signs of ER stress with high levels of CHOP, supporting the rationale for the development of this mouse model, *Chop* deletion in R*yr1*^I4895T^ genetic background does not rescue the perinatal lethality of R*yr1*^I4895T^ or the channel function, but protects cells from ER-stress induced apoptosis.

## Materials and methods

###  Animals

*Ryr1*^I4895T^ mice in the C57BL/6 background were imported from the laboratory of Prof. Susan Hamilton (Baylor College, Houston, Texas) and crossed with *Chop*^*−/−*^ mice in the C57BL/6 background already available in our laboratory. Genotyping at the *Chop* and *Ryr1*^I4895T^ locus followed published procedures^10,16^. To determine the sex of the newborn pups, we employed a multiplex PCR to detect the male-specific sequence Sry together with IL3 in DNA extracted from pups at birth. Primers Sry F: TGGGACTGGTGACAATTGTC and R: GAGTACAGGTGTGCAGCTCT. Primers IL3 F: GGGACTC-CAAGCTTCAATCA and R: TGGAG-GAGGAAGAAAAGCAA (synthesized by Life Technologies/Gibco-BRL, Rockville, MD) as in^19^.

All experimental protocols were approved by the Mario Negri Institute licensing committee. Procedures involving animals and their care were conducted in conformity with the principle of ARRIVE 2.0 and the laws, regulations and policies governing the care and use of laboratory animals: Italian Governing Law (D. lgs 26/2014, authorization number 19/2008-A issued 6 March 2008 by Ministry of Health; 1043/2020-PR to E. Zito); Mario Negri Institutional Regulations and Policies providing internal authorization for people conducting animal experiments (Quality Management System Certificate—UNI EN ISO9001: 2008—registration number 6121); the NIH Guide for the Care and Use of Laboratory Animals (2011 edition); EU directives and guidelines (EEC Council Directive 2010/63/UE).

###  Real-time quantitative RT-PCR analysis

RNA was isolated from leg muscles of newborn pups and from mouse embryonic fibroblats (MEFs) using the RNeasy Mini Kit (Qiagen), reverse-transcribed and analyzed using the Applied Biosystems’ real-time PCR System and the ∆∆Ct method. Relative gene expression in cells was normalized to GAPDH mRNA levels. The primer sequences are described in^16^. Sequence of Ryr3 primers were Ryr3F: ACCAGCAGG AGCAAG TACG, Ryr3R: GGGGTCGTGTCAAAGTAGTCA.

### MEFs

MEFs with RyR1^IT^ or CHOP mutation were isolated on embryonic day (E.) 13.5 and studied as primary MEFs. MEFs were also immortalized after serial passages following transfection with SV-40 large T antigen and cultured in DMEM supplemented to 25 mM glucose, 10% FCS and non-essential amino acid. ERO1 alpha knock out MEFs were characterized in^20^.

### Western blotting

Protein concentration was determined with a standard BCA assay (Pierce). Samples with the same protein concentration were mixed with non-reducing Laemmli buffer (62.5 mM Tris–HCl pH 6.8, 2% SDS, 10% glycerol and 0.01% bromophenol blue), supplemented with 100 mM DTT and heated for 5 min at 95 °C. Protein samples separated by reducing SDS-PAGE were transferred to Protran nitrocellulose membrane (GE10600002, Amersham Protran, pore size 0.45 μm) and probed with monoclonal mouse anti-Actin (MAB1501, Sigma Aldrich) polyclonal rabbit anti-ERO1 alpha^21^. The fluorescent secondary antibodies IRDye 680RD goat anti-mouse IgG (926–68,070, Li-Cor) and IRDye 800CW goat anti-rabbit IgG (926–32,211, Li-Cor) were used for protein detection. The fluorescent signal was acquired on a ChemiDoc MP Imaging System and quantified by Image Lab analysis software (Bio-Rad laboratories).

### MTS assay

Three thousand cells were incubated in MTS [3-(4,5-dimethylthiazol-2-il)-5-(3-carboxymethoxyphenjl)-2-(4-sulfophenjl)-2H-tetrazolio] and PMS (phenazine methosulfate), as indicated in the CellTiter 96^®^ Aqueous Non-Radioactive Cell Proliferation Assay (Promega). For acquisition, we used TECAN infinite M200 with excitation wavelengths at 490 nm.

### FACS analysis

DNA flow cytometric analyses were performed on 5 × 10^4^ cells labeled with Annexin V-FITC and propidium iodide (Invitrogen) at the acquisition rate of 300 events per second, using a CytoFLEX LX flow cytometer (Beckman Coulter, Brea, CA, USA).

### Chemicals

Thapsigargin (Tg, Santa Cruz Biotechnology Inc.) and Tunicamycin (Tun, Sigma Aldrich) were incubated on cells for 24 h at the concentrations indicated. Caffeine (Cf), ATP, 2-Aminoethoxydiphenyl borate (2-APB), ryanodine (Ry) and Ru360 were purchased from Sigma-Aldrich (Milan, Italy). Fluo-4-acetoxymethyl ester and Rhod 2-acetoxymethyl ester were purchased from Thermo Fisher Scientific (Milan, Italy). The cells were loaded with the two probes, then incubated for 10 min with 10 mM Cf or 1 mM ATP with or without 50 µM 2-APB (2-aminoethoxydiphenyl borate, an inhibitor of IP3R), 100 µM Ry (ryanodine, an inhibitor of RYR) or 10 µM Ru360 (an inhibitor of mitochondrial calcium uptake).

### Measurement of cytosolic and mitochondrial Ca^2+^

MEFs grew in 35 mm tissue culture dishes with an uncoated coverslip, treated for 20 min with 4 µM Fluo 4-AM (a fluorescent probe for the detection of cytosolic calcium) or 10 µM Rhod 2-AM (a fluorescent probe for the detection of mitochondrial calcium), then exposed for another 10 min to the IP_3_R or RyR agonists. The cells were finally washed three times in phosphate buffer saline (PBS; 136 mM NaCl, 10 mM Na_2_HPO_4_, 1.5 mM KH_2_PO_4_, 3 mM KCl; pH 7.4) and fluorescence images were collected with a BX-51 microscope (Olympus, Milan, Italy), equipped with a SPOT-RT camera unit (Diagnostic Instruments, Delta Sistemi, Rome, Italy) using an Olympus LCAch 40 × /0.55 objective lens. The excitation and emission wavelengths were set at 488 and 515 nm for Fluo 4, and at 540 and 590 nm for Rhod 2 with a 5-nm slit width for both emission and excitation. Images were collected with exposure times of 100–400 ms, acquired digitally and processed for fluorescence determination at the single cell level, with ImageJ software. Mean fluorescence was determined by averaging the fluorescence values of at least 50 cells/treatment condition/experiment.

### Statistics

Data are the mean ± SEM, and were analyzed with Prism 7 (Graphpad). Statistical significance was established using the unpaired t-test for two-group analysis and one-way ANOVA multiple comparison tests for three or more groups. One asterisk indicates *p* < 0.05, two for *p* < 0.01, three for *p* < 0.001 and four for *p* < 0.0001.

## Results

### Compound homozygous ***Ryr1***^I4895T/I4895T^, ***Chop***^−***/***−^ mice die at birth

**Figure 1:**
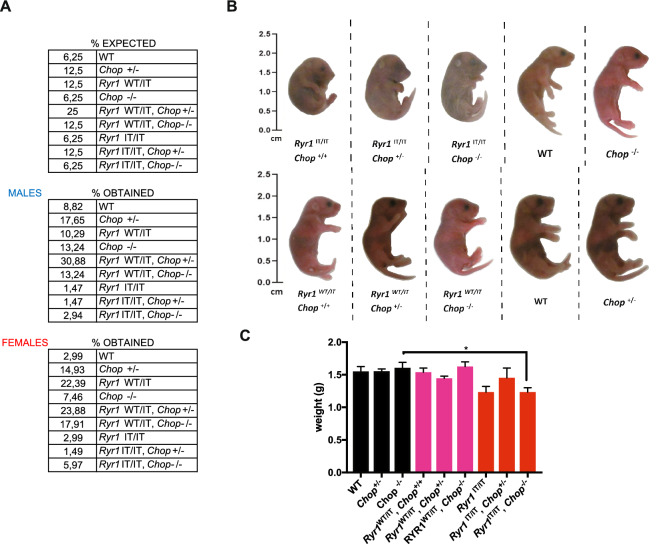
Low recovery rate and gross morphology of *Ryr1*^I4895T/I4895T^, *Chop*^*−/−*^ newborn pups. (**A**) The expected and observed distribution of males and females, as percentages of genotypes on day one among a progeny of one hundred thirty-five (sixty-eight males and sixty-seven females) newborns pups from a cross between *Ryr1*^*WT*/I4895T^, *Chop*^+*/−*^ trans-heterozygote female and male mice. (**B**) Representative gross lateral view of newborn pups (the vertical black lines indicate that the views of the pups were trimmed from different snapshots from which the original background was erased and the views of the different pups copied on the same background) of the indicated genotypes. *Ryr1*^*I4895T*/I4895T^, *Ryr1*^I4895T/I4895T^, *Chop*^+*/−*^* Ryr1*^I4895T/I4895T^, *Chop*^*−/−*^ have a curved posture. (**C**) Bar graphs depicting the mean weight of newborn mice at birth.

To study the importance of CHOP on the phenotypical consequence of *Ryr1*^I4895T^ we crossed *Ryr1*^WT/I4895T^ mice with *Chop*^**−/−**^ mice. The compound homozygous *Ryr1*^I4895T/I4895T^, *Chop*^**−/−**^ was obtained by crossing *Ryr1*^*WT*/I4895T^, *Chop*^+*/−*^ trans heterozygote mice. Crosses between these trans heterozygotes yielded the compound homozygous *Ryr1*^I4895T/I4895T^, *Chop*^**−/−**^ as well as *Ryr1*^I4895T/I4895T^ and *Ryr1*^I4895T/I4895T^, *Chop*^+*/−*^. Mice were genotyped at birth with previously established procedures^10,16^ and females were distinguished from males by a PCR-based genotyping to detect the male-specific sequence (Sry). However, on a total of one hundred thirty-five pups, all *Ryr1*^I4895T/I4895T^ pups also with mixed *Chop* genotypes were recovered at a lower frequency than that predicted by Mendelian transmission of the mutant alleles. The yield was better for the females with *Ryr1*^I4895T/I4895T^ genotype and mixed *Chop* deletion which was very close to that predicted by Mendelian transmission in *Ryr1*^I4895T/I4895T^, *Chop*^**−/−**^ (Fig. 1A).

All the recovered pups with *Ryr1*^I4895T/I4895T^ genotypes, with or without any *Chop* deletion, died soon after birth while siblings of the other mixed genotypes survived (Fig. 1A). The pups with *Ryr1*^I4895T/I4895T^ genotypes and with or without any *Chop* deletion were readily identifiable at birth among other siblings given their curved posture and lack of any response to stimuli. Soon after birth, these mice became cyanotic and died, probably unable to breathe as previously reported^11^ (Fig. 1B). Mice with *Ryr1*^I4895T/I4895T^ genotypes had lower birth weight but no appreciable difference in weight were seen among mice with *Ryr1*^I4895T/I4895T^ genotypes and mixed *Chop* deletion (Fig. 1C). These findings unequivocally suggest that CHOP deletion does not rescue the perinatal lethality of *Ryr1*^I4895T/I4895T^ mice.

### Skeletal muscle of ***Ryr1***^I4895T/I4895T^ mice shows signs of ER stress

**Figure 2:**
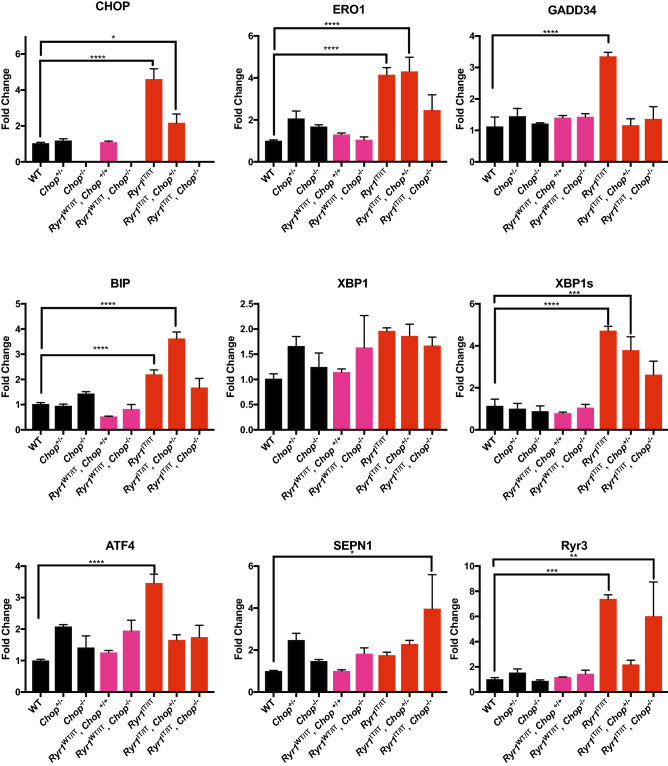
Analysis of ER stress and UPR in skeletal muscle with mixed *Ryr1*^I4895T^ *CHOP* background. Semi-quantitative real-time RT-PCR analysis of ER stress/ER stress response markers (together with *Sepn1* and *Ryr3*) from mRNA from leg muscles of newborn pups of the genotypes indicated (*N* = 3).

Quantitative real-time PCR on cDNA from the leg muscles of the few *Ryr1*^I4895T/I4895T^ pups recovered indicated increased levels of CHOP, ERO1, GADD34, XBP1 spliced, ATF4 and BIP, suggesting ER stress and UPR in their muscle while the levels of these ER stress/UPR markers were low and comparable to those of WT in the skeletal muscle of *Ryr1*^I4895T/WT^. These findings suggest ER stress and UPR in the skeletal muscle of newborn *homozigous Ryr1*^I4895T/I4895T^ but not in *heterozigous Ryr1*^I4895T/WT^ pups. However, while CHOP levels were half in the skeletal mice of *Ryr1*^I4895T/I4895T^, *Chop*^+*/−*^ and abolished, as expected, in *Ryr1*^I4895T/I4895T^, *Chop*^*−/−*^*,* and GADD34 followed the same pattern, the other ER stress/UPR markers ERO1 and BIP did not follow the CHOP patterns, i.e.; ERO1 and BIP were not downregulated in *Ryr1*^I4895T/I4895T^, *Chop*^+*/−*^ but only in *Ryr1*^I4895T/I4895T^, *Chop*^*−/−*^ and SEPN1 was even upregulated in mice with the latter genotype (Fig. 2). These findings support the possibility that *Chop* deletion might lower ongoing ER stress/ER stress response in the skeletal muscle of *Ryr1*^I4895T/I4895T^ pups.

### Chop deletion does not protect ***Ryr1***^I4895T/I4895T^ from the consequences of severe ER stress

**Figure 3:**
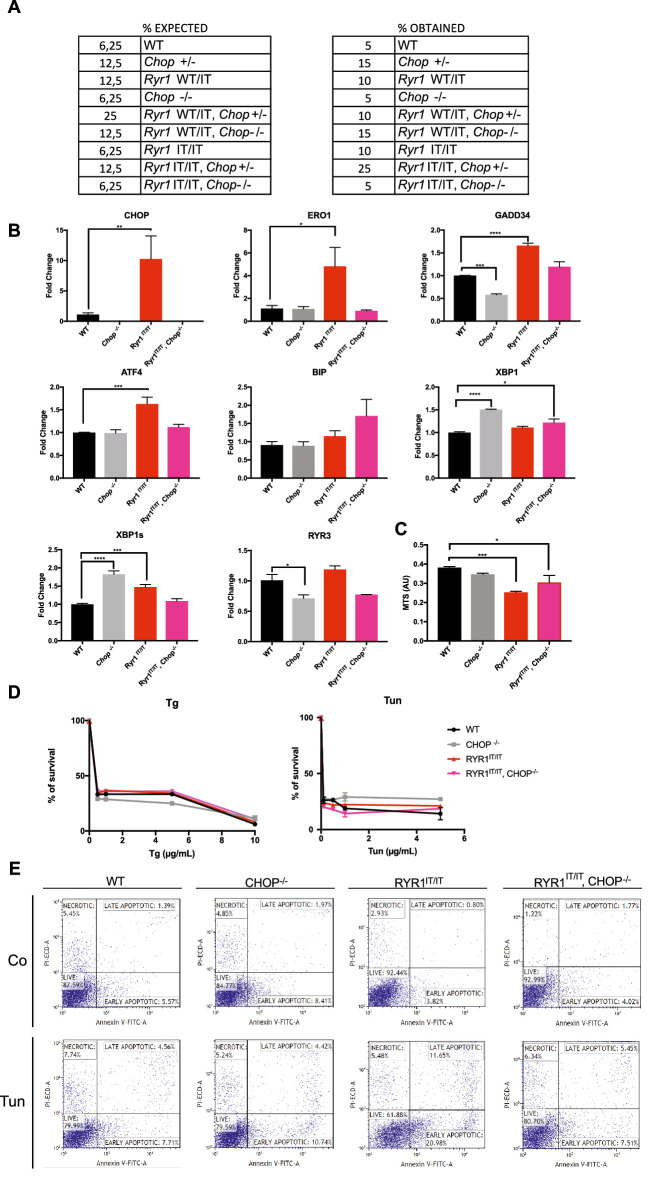
Effect of CHOP deletion on ER stress levels and the viability of *Ryr1*^I4895T/I4895T^ cells. (**A**) The expected and observed distribution of twenty embryos on E13.5 as a percentage of the different genotypes obtained from a cross between *Ryr1*^*WT*/I4895T^, *Chop*^+*/−*^ trans-heterozygote female and male mice. (**B**) Semi-quantitative real-time RT-PCR of ER stress/ER stress response markers (and Ryr3) from mRNA prepared from MEFs of the indicated genotypes (*N* = 4). (**C**) Bar graphs presenting the growth rate (MTS) in 24 h of an equal number of cells with the indicated genotypes (*N* = 5). (**D**) Survival of MEFs that received no treatment or were treated with the indicated concentrations of thapsigargin (Tg) and tunicamycin (Tun) for 24 h. Survival is expressed as the relative amount of MTS signal reduced by thapsigargin- and tunicamycin-exposed cells compared with unexposed cells (arbitrarily set to 100%) (*N* = 5). (**E**) Apoptosis analysis. Cells treated with or without tunicamycin (1μg/mL) were analyzed for Annexin-V and propidium iodide (PI) by flow cytometry and divided into necrotic, late apoptotic, early apoptotic and live cells.

The difficulty of recovering enough live *Ryr1*^I4895T/I4895T^ pups to isolate skeletal myotubes limited our studies on RYR1 activity in skeletal muscle-derived cell cultures. Early reports indicated RYR1 expression in cells different from those of the skeletal muscle, such as the fibroblasts^22^, and indicated early expression of *Ryr1* transcript already at E (embryo day) 9.5^11^. We then looked at MEFs, which are extracted from embryos on E13.5, for functional studies on *Ryr1*^I4895T/I4895T^. There was no detrimental effect of the I4895T mutation at this embryonal stage, and in fact *Ryr1*^I4895T/I4895T^ embryos were recovered at the predicted yield of the Mendelian transmission of the mutant alleles (Fig. 3A).

WT, *Chop*^*−*/*−*^, *Ryr1*^I4895T/I4895T^ and *Ryr1*^I4895T/I4895T^, *Chop*^*−*/*−*^ MEFs were extracted from embryos, genotyped and cultured (Fig. Supplementary 1A). Quantitative real-time PCR on cDNAs from MEFs indicated the presence of *Ryr1* transcript in MEFs, although sixty times less than in the diaphragm (data not shown). We tested also Ryr3, the ubiquitously expressed Ryr isoform, which resulted upregulated in *Ryr1*^I4895T/I4895T^ muscle but not in MEFs (Fig. 2 and Fig. 3B). Therefore, we used MEFs as a surrogate cell system to study whether *Chop* deletion protects *Ryr1*^I4895T/I4895T^ cells from the consequences of ER stress and influences RYR1 activity.

Primary *Ryr1*^I4895T/I4895T^ MEFs, and SV40-immortalized *Ryr1*^I4895T/I4895T^ MEFs had increased CHOP, ERO1, GADD34, ATF4, XBP1 spliced levels, as already seen in the skeletal muscle of mice with the same genotype, but BIP did not increase significantly in these MEFs. Importantly and in line with what was seen in skeletal muscle, ERO1, GADD34, ATF4, XBP1 spliced levels were significantly reduced in *Ryr1*^I4895T/I4895T^, *Chop*^*−/−*^ (Fig. 3B).

*Ryr1*^I4895T/I4895T^ MEFs had reduced cell viability compared to WT, which it was better in *Ryr1*^I4895T/I4895T^ *Chop*^*−/−*^ MEFs, suggesting that there is a positive correlation between reduced ER stress and improved viability in the latter (Fig. 3C). To understand whether lack of CHOP protected *Ryr1*^I4895T/I4895T^ against the consequences of severe ER stress, MEFs underwent treatments with different doses of the two ER stress inducer drugs tunicamycin and thapsigargin and their viability was analyzed. *Ryr1*^I4895T/I4895T^, *Chop*^*−/−*^ MEFs were not protected from the detrimental effect on the viability (as detected by a MTS assay) of the exposure to these two drugs (Fig. 3D). However, the analysis of an apoptotic readout, i.e., the staining Annexin V and propidium iodide, on cells treated for twelve hours with tunicamycin indicated a higher rate of apoptosis in *Ryr1*^I4895T/I4895T^ which was much lower in *Ryr1*^I4895T/I4895T^, *Chop*^*−/−*^ MEFs, suggesting that CHOP deletion preserved *Ryr1*^I4895T/I4895T^ cells from tunicamycin-induced apoptosis (Fig. 3E).

These observations indicate that *Chop* deletion does not protect the overall viability of *Ryr1*^I4895T/I4895T^ MEFs against the consequences of severe ER stress but protects them from ER stress-induced apoptosis.

### RYR1 ^I4895T^ activity is not rescued following CHOP deletion

**Figure 4:**
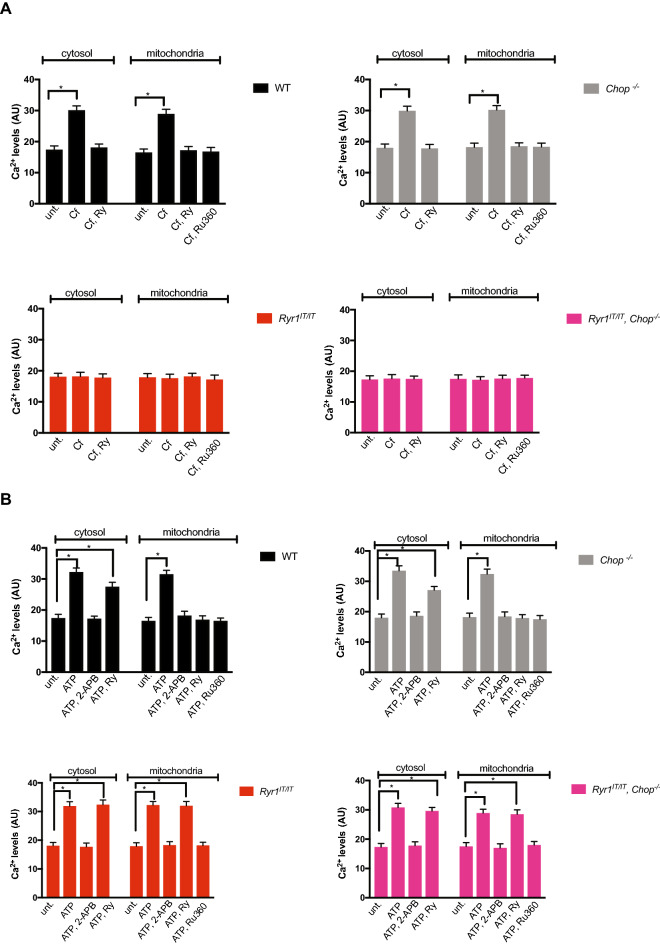
Calcium handling in *Ryr1*^I4895^^T/I4895T^ and in *Ryr1*^I4895T/I4895T^, *Chop*^*−/−*^ cells. (**A**) Bar graphs depicting the effects of caffeine (Cf, an agonist of the RYR), Ryanodine (Ry, an inhibitor of the RYR) and Ru360 (an inhibitor of mitochondrial calcium uptake) on calcium levels in cytosol (detected by the Fluo 4 probe) and in mitochondria (detected by the Rhod 2 probe) in the MEFs of the indicated genotypes. (**B**) Bar graphs depicting the effects of ATP (an agonist of IP3r), 2-APB (an inhibitor of IP3r), Ry and Ru360 on calcium levels in cytosol (detected by the Fluo 4 probe) and in mitochondria (detected by the Rhod 2 probe) in the MEFs of the indicated genotypes. For clarity, the value of untreated cells is duplicated and presented in panels (**A**) and (**B**). Results are the means ± SD calculated from three separate experiments (ANOVA followed by Dunnett's test).

WT, as well as *Chop*^*−/−*^ MEFs, were responsive to the RYR agonist caffeine (Cf)^23^, as suggested by the increases in calcium levels in the cytosol and mitochondria, pointing to a functional RYR in MEFs (Fig. 4A). Consistently with this, the calcium levels were blunted in both organelles by a high concentration of the alkaloid ryanodine (Ry), a RYR antagonist^23^. Mitochondrial calcium accumulation was impaired by Ru360, an inhibitor of the mitochondrial calcium uniporter^24^ (Fig. 4A). In contrast, caffeine did not raise calcium levels in cytosol and mitochondria of *Ryr1*^I4895T/I4895T^ and *Ryr1*^I4895T/I4895T^, *Chop*^*−/−*^ MEFs, suggesting that both MEFs express a non-functional caffeine-insensitive RYR (Fig. 4A).

However, ATP, an IP3 receptor (r) agonist^25^, raised calcium levels in cytosol and mitochondria through a mechanism sensitive to 2-APB, an IP3r antagonist, in all four genotype-divergent MEFs^26^ (Fig. 4B). Interestingly, Ry partially reduced the ATP-dependent increase in cytosolic calcium and suppressed the mitochondrial calcium accumulation in WT and *Chop*^*−/−*^ MEFs, implying the involvement of the RYR in mitochondrial calcium accumulation after IP3r stimulation, as already seen in other RYR expressing cells^27^. However, while Ry had no detectable effects in *Ryr1*^I4895T/I4895T^ and *Ryr1*^I4895T/I4895T^, *Chop*^*−/−*^ MEFs, the combination of ATP with Ry raised cytosolic and mitochondrial calcium in *Ryr1*^I4895T/I4895T^ and *Ryr1*^I4895T/I4895T^, *Chop*^*−/−*^ (Fig. 4B).

Thus, in *Ryr1*^I4895T/I4895T^ MEFs and in their counterpart without CHOP, calcium mobilization was compensated by the involvement of IP3r. Impressed by^28^, which reported a hyperactive effect of the loss of the CHOP mediator ERO1 on the cardiac-selective isoform of RyR, RyR2, we evaluated RyR-dependent calcium transients to caffeine in ERO1-alpha knock-out MEFs. However, ERO1 loss in these cells and in basal conditions did not change neither cytosolic or mitochondrial calcium transients (Fig. Sup. 1B and C).

Altogether, the above pharmacological studies indicate a non-responsive RyR to the agonist caffeine in *Ryr1*^I4895T/I4895T^ MEFs whose activity is not rescued by CHOP deletion.

## Discussion

RYR1-related myopathy is a class of rare muscular diseases due to heterozygous or homozygous mutations in *RYR1,* which encodes the Ca^2+^ channel RYR1. These mutations can result in a leaky, E-C-uncoupling or complete loss of the channel. There is still no treatment for RYR1-RM. Progress has been made for some *RYR1* mutations: Rycals are a novel class of small molecules which restore the calstabin1 binding to RyR1 channel, stabilizing the closed state of the channel and resulting in a potential valid treatment for RYR1-RM with a leaky channel^29^. However, there is still a critical need for studies on the pathogenesis of RYR1-RM to identify treatments for the other mutations such as those that give rise to E-C-uncoupling.

The significant degree of clinical (i.e., muscle weakness, breathing problems, scoliosis) and histopathological (i.e., minicores) overlap between SEPN1-RM and RYR1-RM might reflect molecular defects common to these two genetically distinct pathologies. Although with two distinct functions SEPN1 and RYR1 are both localized in the ER/SR membrane and involved in calcium homeostasis of the ER/SR. Selenoprotein N1 is a type II selenocysteine-containing transmembrane protein of the ER which works as a calcium sensor, activating the Ca^2+^ pump SERCA and protecting it against the excessive oxidation imposed by the ER stress-induced oxidase ERO1 alpha (henceforth ERO1)^17,30,31^. RYR1 is a redox-sensitive Ca^2+^ channel of the ER/SR, that triggers Ca^2+^ efflux from this cellular compartment, leading to excitation–contraction (E-C) coupling^32^. Primary myotubes from patients with either SEPN1 null or RYR1 mutations present oxidative stress which can be rescued by the reductant N-acetylcysteine (NAC)^33^. This result led to the first therapeutic trials with NAC in SEPN1-RM and RYR1-RM (ClinicalTrials.gov NCT02505087 and NCT02362425). However, the RYR1 trial with NAC failed to achieve its primary endpoint, while the SEPN1 results are still under analysis^34^. Thus, new molecular determinants are still needed common to the pathogenesis of these two diseases, which can justify the overlapping symptoms.

Skeletal muscle is exposed to physiological triggers of ER stress, such as hypoxia, reactive oxygen species (ROS) triggered during muscle contraction, and unbalanced Ca^2+^. Thus, skeletal muscle might experience ER stress despite not being a highly secretory tissue (i.e., with a high load of proteins to fold). Furthermore, the response to ER stress, referred to as the Unfolded protein response (UPR), is involved in the physiological function of the skeletal muscle, providing one more proof of the existence of ER stress and its response (UPR) in this tissue^32,35^. Although UPR is first of all a homeostatic response, in some circumstances, still to be completely elucidated, it can be maladaptive, and in fact deletion of one of its mediators, the gene encoding CHOP, preserves tissue function in cases of a pathological ER stress response^12–14^. Importantly, in the case that ER stress and the consequent UPR were pathogenic in skeletal muscle, new molecules that target specific branches of these pathways are available for pharmacological intervention^36^.

Although SEPN1 regulates SERCA and thus ER calcium uptake, while RYR1 regulates calcium release from ER, both SEPN1 and RYR1 mutants show ER calcium defects, which might imply a common underlying mechanism triggering ER stress and maladaptive UPR, giving rise to the overlapping pathological phenotype. In this regard, we found that the ablation of CHOP rescued the muscle phenotype of SEPN1 KO mice while it reduced ERO1 levels in skeletal muscle, suggesting that the CHOP/ERO1 axis might be the main contributor to SEPN1-RM^16,30,37^.

The I4898T mutation is one of the most common *RYR1* mutations in humans. Analysis of myotubes from mice carrying *Ryr1*^I4895T^ showed that the receptor lacks voltage- and 4-CMC-induced Ca^2+^ release, suggesting that it belongs to the class of mutations generating an uncoupling receptor. Skeletal muscles of *Ryr1*^I4895T/WT^ mice point to altered Ca^2+^ handling, increased ROS, ER stress/UPR, with the upregulation also of CHOP and ERO1, associated with defective muscle force^10^. Importantly, the ER stress inhibitor and chemical chaperone 4-PBA rescues the pathological muscle phenotype of *Ryr1*^I4895T/WT^ mice raising the possibility that ER stress is an important driver also for this myopathy.

On the basis of this rationale, we generated mice with genetic deletion of *Chop* in *Ryr1*^I4895T^ genetic background to study whether ablation of CHOP and thus lack of a maladaptive component of UPR might protect from the pathological consequences of *Ryr1*^I4895T^.

*Ryr1*^I4895T/I4895T^ newborn mice with mixed *Chop* genotypes were recovered at a lower frequency than that predicted by Mendelian transmission of the mutant alleles, which might reflect missed animals on day one rather than embryonal lethality of the mutation. Indeed, the rare newborns with *Ryr1*^I4895T/I4895T^ mutation and mixed *Chop* genotypes were recovered only when we assisted to the delivery and they died soon after. Unfortunately, the lack of CHOP, although it attenuates ERO1, GADD34 and the chaperone BIP levels in skeletal muscle of *Ryr1*^I4895T^ mice, suggesting reduced ER stress, does not recover RYR1 activity or rescue the perinatal lethality of *Ryr1*^I4895T^ mice. This suggests that the failure in the recovery of any RYR1 activity frustrates the rescue of the perinatal lethality despite the reduced ER stress.

This study highlights the untoward consequences of the lack of a mediator of the maladaptive branch of UPR in the missed rescue of *Ryr1*^I4895T^ muscle phenotype. Thus, this study excludes the ER stress/maladaptive branch of UPR, or at least its mediator CHOP, as the primary cause of this RYR1-RM. Further studies will aim to clarify whether CHOP deletion in a genetic background with a more modest increase in *Ryr1*^I4895T^, as in *Ryr1*^I4895T/WT^, triggers any increase/change in RYR1 activity and rescues the related less severe muscle pathological phenotype.

## Supplementary Information

Supplementary Information.

## Data Availability

All data generated or analysed during this study are included in this published article.
