# Supplementary material for: Loss-of-rescue of Ryr1I4895T-related pathology by the genetic inhibition of the ER stress response mediator CHOP
Source: Sci Rep. 2022 Nov 30;12:20632. doi: 10.1038/s41598-022-25198-y (PMC9712496; doi:10.1038/s41598-022-25198-y)
Supplement: Supplementary file 1 — Supplementary Information. [file 41598_2022_25198_MOESM1_ESM.pdf]

Figure Supplementary 1

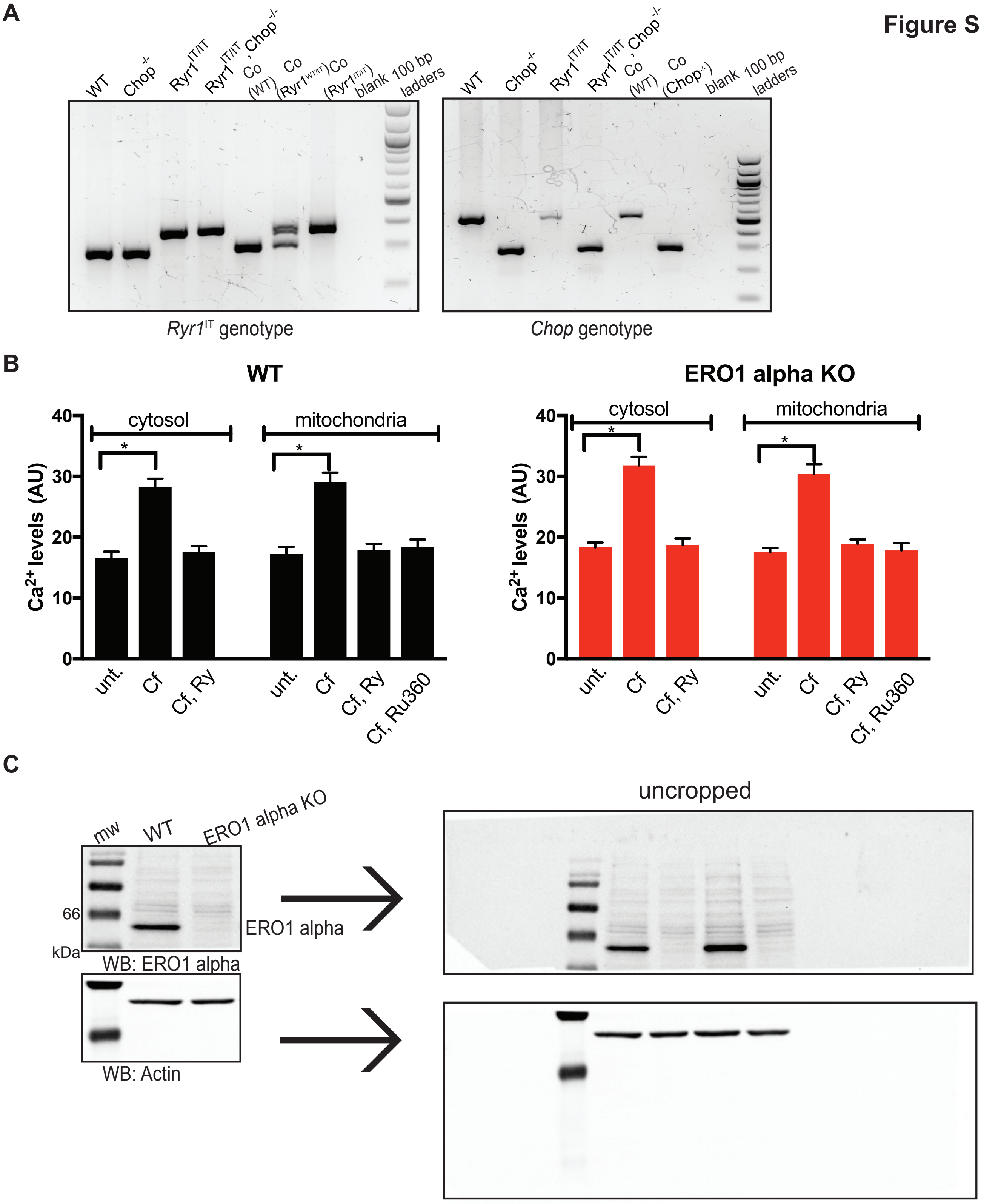

Figure Supplementary 1

A) Agarose gels representing *RyR*^{IT} and *Chop* genotypes of MEFs (100 bp DNA ladder). B) Bar graphs depicting the effects of caffeine (Cf, an agonist of the RYR), Ryanodine (Ry, an inhibitor of the RYR) and Ru360 (an inhibitor of mitochondrial calcium uptake) on calcium levels in cytosol (detected by the Fluo 4 probe) and in mitochondria (detected by the Rhod 2 probe) in WT and ERO1 alpha knock out (KO) MEFs. Results are the means ± SD calculated from three separate experiments (ANOVA followed by Dunnett's test). C) ERO1 alpha and Actin Immunoblot on WT and ERO1 alpha KO MEFs (mw: molecular weight).
